# Congenital Chagas Disease in the United States: Cost Savings through Maternal Screening

**DOI:** 10.4269/ajtmh.17-0818

**Published:** 2018-04-30

**Authors:** Eileen Stillwaggon, Victoria Perez-Zetune, Stephanie R. Bialek, Susan P. Montgomery

**Affiliations:** 1 Department of Economics, Gettysburg College, Gettysburg, Pennsylvania;; 2 International Finance Division, Board of Governors of the Federal Reserve System, Washington, District of Columbia;; 3 Parasitic Diseases Branch, Division of Parasitic Diseases and Malaria, Center for Global Health, Centers for Disease Control and Prevention, Atlanta, Georgia

## Abstract

Chagas disease, caused by *Trypanosoma cruzi*, is transmitted by insect vectors through transfusions, transplants, insect feces in food, and from mother to child during gestation. Congenital infection could perpetuate Chagas disease indefinitely, even in countries without vector transmission. An estimated 30% of infected persons will develop lifelong, potentially fatal, cardiac or digestive complications. Treatment of infants with benznidazole is highly efficacious in eliminating infection. This work evaluates the costs of maternal screening and infant testing and treatment of Chagas disease in the United States. We constructed a decision-analytic model to find the lower cost option, comparing costs of testing and treatment, as needed, for mothers and infants with the lifetime societal costs without testing and the consequent morbidity and mortality due to lack of treatment or late treatment. We found that maternal screening, infant testing, and treatment of Chagas disease in the United States are cost saving for all rates of congenital transmission greater than 0.001% and all levels of maternal prevalence above 0.06% compared with no screening program. Newly approved diagnostics make universal screening cost saving with maternal prevalence as low as 0.008%. The present value of lifetime societal savings due to screening and treatment is about $634 million saved for every birth year cohort. The benefits of universal screening for *T. cruzi* as part of routine prenatal testing far outweigh the program costs for all U.S. births.

## INTRODUCTION

Chagas disease, also called American trypanosomiasis, is caused by *Trypanosoma cruzi*, which is spread by triatomine insect vectors.^1,2^ Infected insects are found from southern South America to the southern states of the United States, although vector transmission to humans is very rare in the United States.^3–5^ Vector control has been very successful in many endemic regions.^1,2,6^ Transmission can also occur through blood transfusions, organ transplantation, consumption of insect feces in food, and from mother to child during gestation.^3^ Almost all endemic countries have instituted screening of blood products.^1,2^ Despite the existence of domestic and wild animal reservoirs, significant progress in reducing incidence is possible through rigorous domestic vector control and screening of blood and organ donors.^1^ Congenital transmission, on the other hand, could perpetuate the existence of Chagas disease indefinitely, even in countries or regions with no or almost no autochthonous vector transmission.^7–10^ This article uses a decision-analytic model to evaluate the societal economic cost of maternal screening to identify and treat infected newborns and their mothers compared with the societal cost of undiagnosed or late-diagnosed Chagas disease in the United States.

### Prevalence.

The World Health Organization estimates that there are 5.7 million people infected with *T. cruzi* in Latin America^1^ and about 400,000 Latin Americans with Chagas disease residing in Europe, Japan, and the United States.^4,6,11^ Successful programs in domestic and peridomestic vector control reduced the estimated number of new cases from 700,000 per year in the 1970s to 40,000 per year in 2010.^1,6^ Vector transmission has been the predominant mode, but with control programs and blood bank screening, incidence is decreasing. Congenital transmission, however, accounts for 9,000 new cases per year in Central and South America^1^ and several hundred more in the United States and Europe.^4,12^ The Pan American Health Organization estimates that a quarter of new cases are caused by congenital transmission.^7,13^

### Nature of the disease.

Chagas disease has two stages—acute and chronic.^2,6,14^ In the acute phase, most newly infected persons, including children infected by insect vectors and infants infected congenitally, are asymptomatic or display nonspecific symptoms.^6,14^ Infants with congenital infection may present with fever, anemia, and low Apgar scores—symptoms that can lead to misdiagnosis at birth of toxoplasmosis, rubella, or cytomegalovirus.^6,10,15^ In a small number of cases, congenitally infected infants will have severe symptoms, including meningoencephalitis, gastrointestinal complications, and respiratory distress.^3,15,16^ In most newly infected persons, the acute phase lasts 4–12 weeks, during which parasitemia is high.^3,6^

Following the acute phase, infected persons enter the indeterminate form of chronic phase infection, which can last 10–30 years or an entire lifetime.^3,6^ Precisely defined, persons with the indeterminate form of chronic Chagas disease have “positive anti-*T cruzi* serology, no symptoms or physical examination abnormalities, normal 12-lead electrocardiogram (ECG) findings, and normal findings on radiological examination of the chest, esophagus, and colon” (page 2172),^14^ see also ref. 6. An estimated 60–70% of infected persons will remain in this form for the rest of their lives and will have normal life expectancy.^6^

The proportion of chronically infected persons who will begin to show symptoms, that is, enter the chronic determinate form, has been estimated from 30% to 40%, generally from 10 to 30 years after infection.^1,6^ Symptoms can be mild and may not be identified as Chagas related. For some, however, the first recognized sign of Chagas disease is sudden cardiac death.^6^ Chagas disease is the leading cause of heart disease among persons living in poverty in Latin America.^13^ Cardiovascular symptoms of Chagas disease include conduction system abnormalities, arrhythmias, and heart failure.^14^ Digestive symptoms of Chagas disease occur less often and include megacolon and megaesophagus.^6^ Chagas disease has the highest disease burden of all parasitic diseases in the Americas,^2^ and the human cost of premature death and disability due to Chagas disease is substantial. The economic cost of Chagas disease is also high, including the loss of workers through death and disability, as well as the costs of medical interventions for cardiac and digestive pathology. One of the few studies of the economic cost of Chagas disease in Latin America estimated it to be in excess of U.S. $6.5 billion annually, although that was reported in the 1990s, when prevalence was higher.^17^

### Chemotherapeutic treatment.

Currently, two drugs, benznidazole and nifurtimox, are used to treat Chagas disease. In August 2017, benznidazole was approved by the U.S. Food and Drug Administration (FDA) for use in children aged 2–12 years, but as of February 2018, it was not yet available in U.S. pharmacies. As of February 2018, nifurtimox was not FDA approved. Both drugs are currently available under investigational protocols from the Centers for Disease Control and Prevention (CDC).^14^ Efficacy and side effects vary, depending on the age of the person and the length of time since infection.^3,18^ Treatment of infants before the age of 1 year is 90–100% effective in eliminating infection.^12,14^ Estimates of efficacy of treatment of adults range from 40% to 70%.^12^ There is evidence that treating women before pregnancy can reduce the risk of congenital transmission.^19,20^ Treatment has not been shown to reverse cardiac or digestive tract damage among infected persons who already have complications.^21^

### Chagas disease in the United States.

Based on estimates from the U.S. CDC, there are about 300,000 persons with Chagas disease in the United States, the majority of whom were infected in endemic countries before residing in the United States.^4^ Based on the number of women of childbearing age in that population, the birth rate, and the risk of maternal transmission, there are an estimated 63‒315 births of infected infants per year.^4^ There are some local data for pregnant women and newborns. In Texas, among 4,000 women who came for delivery at a Houston hospital serving a mostly immigrant population, 0.25% of mothers had Chagas disease.^22^ Another study found that 0.4% of Hispanic women tested in Houston clinics were infected with *T. cruzi*.^23^

Chagas disease is also transmitted in the United States by insect vectors. A few cases of autochthonous transmission have been reported in the United States.^5,24^ In studies of blood donors, persons with antibody to *T. cruzi* and no history of residence outside the United States have been identified.^25^

### Maternal screening to identify Chagas-infected infants, mothers, and siblings.

Screening of pregnant women and newborns is an accepted protocol for preventing or managing a number of diseases that can be transmitted congenitally, including syphilis, human immunodeficiency virus (HIV), and in some places, toxoplasmosis, rubella, and cytomegalovirus.^8,22,23^ There are as many as 80 newborn screening tests available in some U.S. states, primarily for genetic disorders, but also including also congenital toxoplasmosis and HIV (http://www.babysfirsttest.org/newborn-screening/states).

Because complications in the chronic stage of Chagas disease are associated with long duration of infection,^6^ early diagnosis and treatment are essential not only for newborns but also for their mothers, siblings, and other family members. Pregnant women and babies present a good access point for diagnosing and treating the whole family because prenatal checks and hospital births may afford the greatest likelihood over the lifetime of identifying the affected population, particularly among people who may be reluctant to seek medical attention.

At present, there is no systematic screening for Chagas disease in pregnant women in the United States. Surveys of obstetricians indicate that few are well informed about the risk of Chagas disease, even those who attend to Hispanic women from endemic areas.^26^ Effective screening for Chagas disease would require educating prenatal and delivery care personnel. The concentration of Chagas disease in first- and second-generation Latinos from endemic countries and in nonmigrants residing in specific parts of the United States suggests that targeted screening would be more efficient in terms of cost, education of obstetricians, and minimizing distress for mothers. On the other hand, targeting may be difficult for clinicians and it might be more feasible to include *T. cruzi* in routine screening protocols for all pregnant women.

A protocol for screening could be established as follows: before or during pregnancy or at birth, women would be offered serologic testing, preferably using a serologic rapid test for antibody to *T. cruzi*, to obtain immediate results.^2,14^ Women with positive rapid antibody test results would be further tested to confirm infection. Babies born to infected mothers would be tested at birth (cord blood) and, if negative, again at 4‒6 weeks of life by polymerase chain reaction (PCR) testing, which is the recommended approach for U.S. testing.^14^ (In Latin America, the practice is generally examination of blood smear and/or microhematocrit concentration method.^27^) Because of varying levels of parasitemia in congenital infection, infants with negative results would be tested by serology at 9 months of age or older, after maternal antibody is no longer present.^2,3^ Infected infants would be treated with benznidazole or nifurtimox. Infected mothers would potentially be treated, after ending breastfeeding.^14,15^ Older children of infected mothers would also be tested and treated, if infected. Ideally, other infected family members, such as siblings of the mother, could be identified for treatment and follow-up.

### Economic evaluation of maternal screening.

There have been few studies of the economic cost of Chagas disease and of interventions to reduce morbidity and mortality. Even fewer are the economic evaluations of maternal screening programs. Sicuri and others estimated the costs of a systematic screening program for Latin American women in Spain. They used two decision trees, one for the costs for mothers and another for the costs for infants. They found that a screening program was cost-effective over a broad range of screening costs and probabilities of maternal prevalence and mother-to-child transmission.^12^

## METHOD

We compared the costs of maternal screening and infant testing and treatment as needed (referred to as the Screening option), with the costs of No Screening, with its consequent morbidity and mortality due to lack of treatment or late treatment, using one tree that shows the combined costs for each possible maternal–child pairing. We constructed a decision-analytic model for the United States using TreeAge software (TreeAge Software Inc., Williamstown, MA) to find the lower cost option. The method is the same whether one is evaluating a targeted or a universal screening program because costs and benefits (cost savings) are reported on an individual basis. In the Discussion, we compare the implementation costs and benefits for targeted and universal screening.

The tree comprises a decision node (No Screening or Screening), chance nodes (probabilities of maternal infection, transmission, and various degrees of injury), and terminal nodes for each outcome (sums of costs attributable to each outcome). We use a societal perspective. Each outcome represents the expected value of all economic costs based on a series of conditional probabilities for each branch.

### Probabilities.

Table 1 shows the probabilities of maternal infection (prevalence among women of childbearing age), maternal transmission, conversion from indeterminate form to symptomatic form, and other risks of morbidity and mortality. Point estimates and ranges are derived from the literature indicated in the source column. For prevalence, the point estimate (0.0131) refers to the population of U.S. Hispanic women from endemic areas,^4^ which is appropriate for a model of targeted screening. The range, used in sensitivity analysis (0.0‒0.0131), includes the prevalence among all U.S. women (0.0016), and is thus appropriate for universal screening.

**Table 1:** Probabilities

Nodes	Name in tree	Probabilities	Point estimate (range)	Sources point estimate (range)
2, 85	Prevalence	Maternal prevalence	0.0131 (0–0.0131)	4 (22,23,36)
4, 87	MTCT	MTCT	0.05 (0–0.05)	6,16,28 (3,8,15,36,37)
5, 42, 43, 52, 61, 69, 77	SymptomChagas	Risk of symptomatic Chagas	0.30 (0.2‒0.4)	13,38,39 (3,6,12,17,40^–^46)
7, 18, 27, 36, 45, 51, 54, 63, 71, 79, 91, 102, 111, 120, 129	Cardiac	Of which cardiac	0.67 (0.67‒0.75)	6,45,47 (40)
	#	Of which digestive	0.33 (0.25‒0.33)	6,45,47 (40)
9, 20, 29, 38, 47, 56, 60, 65, 73, 81, 93, 104, 113, 122, 131	CardiacMild	Cardiac mild	0.4 (0.36‒0.44)	48
	CardiacSevere	Cardiac severe	0.3	21
	#	Cardiac very severe	0.3	21
13, 97	SymptomAtBirth	Risk of baby symptomatic at birth	0.1	16,29
14, 98	SymptomAtBirthMild	Mild symptomatic at birth	0.5	16
	SymptomAtBirthSevere	Severe symptom at birth	0.45	16
	#	Neonatal death	0.05	3,6,17,49
15, 24, 33, 88, 99, 108, 117, 126	MotherCured	Treated mother cured	0.6 (0.4‒0.7)	3,12 (12)
	#	(1-MotherCured) × (1-SymptomChagas)	0.28	6
	MotherSymptomAfterRx	(1-MotherCured) × SymptomChagas	0.12 (0.06‒0.24)	6

MTCT = mother-to-child transmission.

In a decision analysis, probabilities are conditional on prior probabilities. For each category, the possibilities are exhaustive (sum to 1.0) and mutually exclusive. For example, as seen in Table 1, of persons infected with Chagas disease, 30% are estimated to have symptoms, of whom about 2/3 will have cardiac symptoms. Similarly, Table 1 indicates that 10% of infected infants are estimated to be symptomatic at birth, and the expected distribution of severity of symptoms among that 10% is given in the subsequent rows of Table 1. The Supplemental Text contains detailed explanation of the derivations and sources.

### Costs.

Table 2 lists all of the unit costs used in the decision tree, including costs of testing and chemotherapy for infants and mothers, costs of hospital care for symptomatic newborns, and costs of interventions for cardiac and digestive tract morbidity. The Supplemental Text contains detailed explanation of the cost derivations and sources.

**Table 2:** Unit costs

Item (name in tree)	Unit cost in 2016	Source
Infant PCR × 2 (Dx_baby)	$400	http://www.parasitic.com/test.htm
Adult ELISA + IFA (Dx_mom)	$60	https://ncifrederick.cancer.gov/programs/science/csp/ltsELISA.asp
		https://www.scienceexchange.com/services/immunofluorescence
		http://www.abcam.com/chagas-igg-elisa-kit-ab178637.html
		https://tvmdl.tamu.edu/?s=Chagas&species=&post_type=tests&test-submit=Search+Tests
		https://www.biocompare.com/pfu/110627/soids/2-399758/ELISA_Kit/ELISA_Trypanosoma_cruzi
Benznidazole, baby, mom (Rx_baby, Rx_mom)	$0	Available without cost from CDC
General practitioner examination, biennial*	$166	http://meps.ahrq.gov/mepsweb/data_files/publications/st484/stat484.shtml
Cardiologist examination, annual*	$303	http://meps.ahrq.gov/mepsweb/data_files/publications/st484/stat484.shtml
ECG + stress test, annual or biennial*	$220	Federal Register 81(219) Nov 14, 2016, page 79724 https://www.gpo.gov/fdsys/pkg/FR-2016-11-14/pdf/2016-26515.pdf
Amiodarone, first year	$425	http://reference.medscape.com/drug/pacerone-cordarone-amiodarone-342296
Annual*	$100	https://data.medicaid.gov/Drug-Prices/NADAC-as-of-2017-01-18/e97y-sprb
Pacemaker*	$22,230	http://medicarehelp.org/cost-of-medicare/procedure/state/Maryland
Heart transplant*	$942,830	http://www.milliman.com/uploadedFiles/insight/Research/health-rr/1938HDP_20141230.pdf
Gastroenterologist, annual*	$310	http://meps.ahrq.gov/mepsweb/data_files/publications/st484/stat484.shtml
Esophageal relaxants, annual*	$160	http://www.mayoclinic.org/diseases-conditions/esophageal-spasms/basics/treatment/con-20025653
		https://data.medicaid.gov/Drug-Prices/NADAC-as-of-2017-01-18/e97y-sprb
Laxatives, annual*	$33	https://www.drugs.com/pro/docusate-sodium.html
		https://data.medicaid.gov/Drug-Prices/NADAC-as-of-2017-01-18/e97y-sprb
Fundoplication*	$11,234	http://www.medicarehelp.org
Colon resection*	$44,718	http://www.medicarehelp.org
Neonatal hospital costs, mild symptoms (CostSympMild)	$2,600	https://www.hcup-us.ahrq.gov/reports/statbriefs/sb113.pdf
Neonatal hospital costs, severe symptoms* (CostSympSev)	$3,900	https://www.hcup-us.ahrq.gov/reports/statbriefs/sb113.pdf

CDC = Centers for Disease Control and Prevention; ECG = electrocardiogram. See Supplement Text for detailed cost derivations; ELISA = enzyme-linked immunosorbent assay; IFA = immunofluorescence assay.

* Part of indeterminate, cardiac, or digestive package (see Table 3 and Supplemental Table 3).

Table 3 lists the present value of lifetime costs of typical protocols for possible outcomes, including the indeterminate form and cardiac and digestive complications of the chronic stage, including productivity losses due to premature mortality. The Supplemental Text contains detailed explanation of the protocols, cost derivations, and sources.

**Table 3:** Conditions and package costs (present value)

Condition	Mothers with *Trypanosoma cruzi* diagnosis	Mothers without *T. cruzi* diagnosis	Babies as adults without diagnosis
Chronic, indeterminate	**$5,495**	No treatment	No treatment
Chronic cardiac, mild	**$106,938**	**$112,095**	**$39,929**
Chronic cardiac, severe	**$407,315**	**$414,519**	**$146,015**
Chronic cardiac, very severe	**$1,893,955**	**$1,905,848**	**$723,246**
Esophageal, mild	$115,367	$109,727	$39,016
Esophageal, severe	$390,998	$387,771	$139,749
Colon, mild	$112,383	$105,952	$37,514
Colon, severe	$435,716	$433,861	$154,109
Digestive care costs, average of digestive conditions	**$263,616**	**$259,328**	**$92,597**

See Supplement Text for detailed protocols and cost derivations for each condition. Amounts in bolded text are used in the decision tree. Unbolded text indicates intermediate calculations used to derive digestive care costs.

We do not include intangible costs, such as pain or worry, nor do we include the lost time of family care givers or incidental costs, such as transportation.

### The decision tree.

The decision tree, Supplemental Figure 1, is available online and shows the probabilities and cost formulas pertaining to each possible outcome. It begins on the left with the decision, No Screening or Screening. In both branches, mothers have the same risk of infection, which is based on the estimated prevalence among Hispanics from endemic countries in the U.S. population^4^ and the same risk of gestational transmission to the child, based on a range of estimates in endemic and non-endemic locations.^6,16,28^

In the No Screening branch, we assume that mothers who deliver asymptomatic babies and the babies themselves will not be identified as being *T. cruzi* infected, even if they develop a digestive or cardiac condition that requires medical attention at some point in their lives. On the other hand, the birth of a symptomatic baby in the No Screening scenario generates a series of actions that are equivalent to the Screening scenario. Even if those symptoms are mild, we assume the best practice—that the baby will be diagnosed with Chagas disease at birth, the mother’s Chagas disease will be diagnosed, and both will be treated. Each terminal node (at the right) shows the costs incurred for both the mother and child in a mother–child pair.

In the Screening branch, before or during pregnancy or at birth, women would be offered serologic testing, preferably using a serologic rapid test for antibody to *T. cruzi*, to obtain immediate results.^2,14^ Women with positive rapid antibody test results would be further tested to confirm infection. Babies born to infected mothers would be tested at birth (cord blood). Infected infants would be treated with benznidazole or nifurtimox. Infected mothers would potentially be treated, after ending breastfeeding.^14,15^ (Negative results in infants of infected mothers are discussed previously in the protocol.)

The decision tree calculates the cost of each possible outcome (indicated at each terminal node) multiplied by its conditional probability (along each branch), which determines the expected value of each possible mother–child outcome (in U.S. dollars). When comparing the option of Screening to No Screening, the decision with a lower expected value for lifetime costs for all possible outcomes is selected as the dominant (lower cost) option.

Although the point estimates are set for targeted screening at current screening cost of $60 per pregnancy, the model also calculates the costs for universal screening and screening cost as low as $8 per pregnancy through sensitivity analysis.

### Sensitivity analysis.

Published studies of Chagas disease report a range of values for the model’s parameters. We have chosen conservative values—ones that seem to represent a consensus among researchers—and we used sensitivity analysis to test the robustness of the results. We derived the range of values for four of the clinical probabilities used in the sensitivity analysis from evidence in the literature: risk of symptomatic Chagas (0.2–0.4), proportion of symptomatic Chagas that is cardiac (0.67–0.75), with the residual in that case being digestive symptoms (0.25–0.33), and the likelihood of cure among adults after the acute phase (0.4–0.7). These ranges are listed in Table 1, column 4, in parentheses, and their respective sources are shown in Table 1, column 5, in parentheses. For the other variables in Table 1, since they do not have a range reported in published literature, we used ±10%.

The cost saving from a maternal screening program can be expected to be especially sensitive to two clinical variables: prevalence among pregnant women and mother-to-child transmission risk. It is argued by some that mother-to-child transmission should be lower in the United States than in highly endemic areas because reinfection in the latter areas increases parasitemia and transmission risk.^29^ On the other hand, higher parasitemia and higher transmission risk have been reported among mothers not exposed to reinfection, possibly because of loss of immunity.^27^ For both prevalence and maternal transmission, we set zero as the lower bound and set the point estimate as the upper bound, as shown in Table 1.

Costs and cost-to-charge ratios can vary greatly across the country and across payers. Consequently, we used sensitivity analysis to test the robustness of our results to differences in treatment costs for indeterminate, cardiac, and digestive Chagas outcomes, treatment costs for symptomatic newborns, and productivity losses pertaining to the various health states. We used ±10% for the upper and lower bounds for each of the costs. In a screening program for a low-prevalence population, however, the cost of testing can be especially important. Whereas we use the testing cost of $60 for the maternal test ($30 × 2 tests), a new point-of-care diagnostic was approved by FDA in December 2016 for which the expected price will be $4.00.^30,31^ For the sensitivity analysis, we set $4 per test as the lower bound ($8 for two tests) and $30 per test ($60 for two tests) as the upper bound to determine the threshold maternal prevalence for which screening is cost saving and the amount of savings under a range of scenarios.

Another form of sensitivity analysis is tornado analysis, which indicates the factors that have the greatest impact on total costs, showing the variation around the expected value for the Screening or No Screening option that results from varying each parameter. An incremental tornado analysis combines the information for both options and determines how the expected value of savings varies because of differences in each parameter. We use the incremental tornado analysis to show the factors that have the greatest impact on expected lifetime savings.

## RESULTS

Supplemental Figure 2, available online, shows the decision tree after calculation. The analysis concluded that Screening is the optimal option. If no screening takes place, the expected value of discounted lifetime costs for all mother–child pairs is $2,321 per pregnancy. The Screening option has an expected cost for infant and mother of $997 per pregnancy. Screening would result in a saving of $1,324 per birth or $636 million for each birth year cohort of 480,000 births to women from endemic areas. These results correspond to the savings per birth in a targeted screening program, using prevalence of 0.0131 among Hispanic women from endemic areas. Table 4 displays these results. This finding lends substantial economic support to a policy of educating obstetricians in the United States and adding maternal screening for Chagas disease to the panel of tests for Hispanic women who themselves or whose mothers lived in endemic areas. (For a description of how costs are calculated for an individual mother–child pair, see Supplemental Text.)

**Table 4:** Results: lifetime societal costs and savings for No Screening and Screening

Scenario	$60 screening cost per birth		$8 screening cost per birth	
	Per birth	Per birth year cohort	Per birth	Per birth year cohort
Targeted Screening				
No Screening	$2,321	$1,114,080,000	$2,321	$1,114,080,000
Screening	$997	$478,560,000	$945	$453,600,000
Savings	$1,324	$635,520,000	$1,376	$660,480,000
Universal Screening				
No Screening	$279	$1,116,400,000	$279	$1,116,400,000
Screening	$172	$690,640,000	$121	$482,640,000
Savings	$106	$425,760,000	$158	$633,760,000

### Sensitivity analyses.

**Figure 1.**
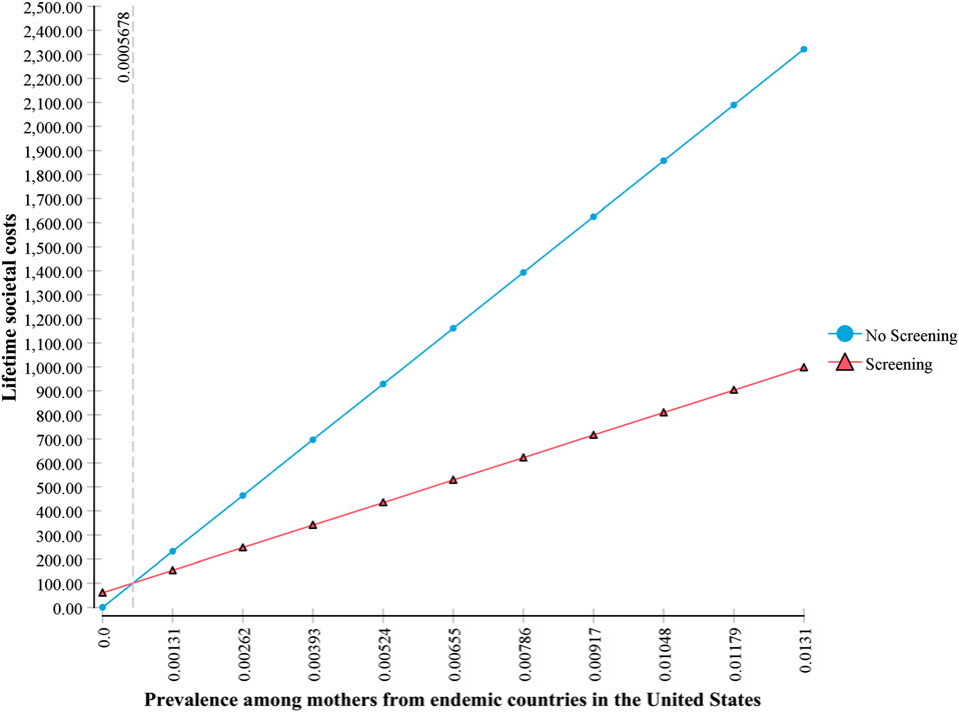
One-way sensitivity analysis on maternal prevalence, showing threshold. This figure appears in color at www.ajtmh.org.

Whereas the point estimates refer to a targeted strategy, the same model demonstrates that universal Screening is also cost saving compared with No Screening at the current screening cost of $60 per pregnancy. The threshold prevalence for which screening is cost saving is 0.0005678, or 0.06%, as shown in Figure 1. The estimated prevalence of *T. cruzi* infection among all 4 million women giving birth per year in the United States is 0.001575, or 0.16%. The savings per birth are lower at lower prevalence but they are still substantial for universal screening. The lifetime costs per birth in the No Screening scenario are $279, and in the universal Screening scenario at the maternal prevalence of 0.16% (estimated U.S. prevalence) and $60 screening cost, lifetime costs are $173 per birth, with savings per birth at $106. For 4 million births annually, that amounts to total savings of $426 million for every birth year cohort.

Availability of a low-cost point-of-care test makes screening an even more viable option. Using $4 per test (× 2 tests = $8 per birth) for maternal diagnosis, we performed a one-way sensitivity analysis on maternal prevalence. At that test cost, the threshold for screening as the cost saving option is maternal prevalence of 0.0000757 (0.008%). In the targeted Screening scenario, savings per birth are $1,376 and total savings for the birth year cohort amount to $660 million.

To get a more detailed look at costs and savings in a universal screening scenario, we show the results of a two-way sensitivity analysis, varying prevalence from 0.0 to 0.001575, the estimated prevalence among all U.S. women in a birth year cohort, and varying the screening cost from $8 per birth to $60 per birth. Table 5 shows the results with five intervals for each parameter. At any screening cost below $40 per birth, the Screening option is cost saving for any nonzero prevalence shown as low as 0.00032, which is 1/5 the actual estimated prevalence among all women giving birth annually in the United States (all non-bolded results). As seen also in Figure 1, universal screening is cost-saving even at $60 per mother, even if national prevalence were far below present estimates.

**Table 5:** Comparison of lifetime costs per birth by prevalence and screening cost

		Screening cost per birth					
		$8.00	$18.40	$28.80	$39.20	$49.60	$60.00
Prevalence		Lifetime costs per birth by prevalence and screening cost (U.S. $)					
0.0	No Screening	0.00	0.00	0.00	0.00	0.00	0.00
	Screening	8.00	18.40	28.80	39.20	49.60	60.00
	Saving	***−8.00***	***−18.40***	***−28.80***	***−39.20***	***−49.60***	***−60.00***
0.000315	No Screening	55.82	55.82	55.82	55.82	55.82	55.82
	Screening	30.53	40.93	51.33	51.33	72.13	82.53
	Saving	*25.29*	*14.89*	*4.49*	*4.49*	***−16.31***	***−26.71***
0.00063	No Screening	111.64	111.64	111.64	111.64	111.64	111.64
	Screening	53.06	63.46	73.86	84.26	94.66	105.06
	Saving	*58.58*	*48.18*	*37.78*	*27.38*	*16.98*	*6.58*
0.000945	No Screening	167.46	167.46	167.46	167.46	167.46	167.46
	Screening	75.60	86.00	96.40	106.80	117.20	127.60
	Saving	*91.87*	*81.47*	*71.07*	*60.67*	*50.27*	*39.87*
0.00126	No Screening	223.28	223.28	223.28	223.28	223.28	223.28
	Screening	98.13	108.53	118.93	129.33	139.73	150.13
	Saving	*125.16*	*114.76*	*104.36*	*93.96*	*83.56*	*73.16*
0.001575	No Screening	279.10	279.10	279.10	279.10	279.10	279.10
	Screening	120.66	131.06	141.46	151.86	162.26	172.66
	Saving	*158.45*	*148.05*	*137.65*	*127.25*	*116.85*	*106.45*

Numbers in italic are savings per birth. Numbers in bold italic are negative savings per birth (losses).

The expected value of the lifetime savings from universal screening with the new point-of-care test (at $8 per birth) is $158 per birth, amounting to $634 million for each birth year cohort, as shown in Table 4. The sensitivity analysis demonstrates that universal screening is cost saving over a wide range of prevalence and screening costs in the United States.

### Incremental tornado analysis.

**Figure 2.**
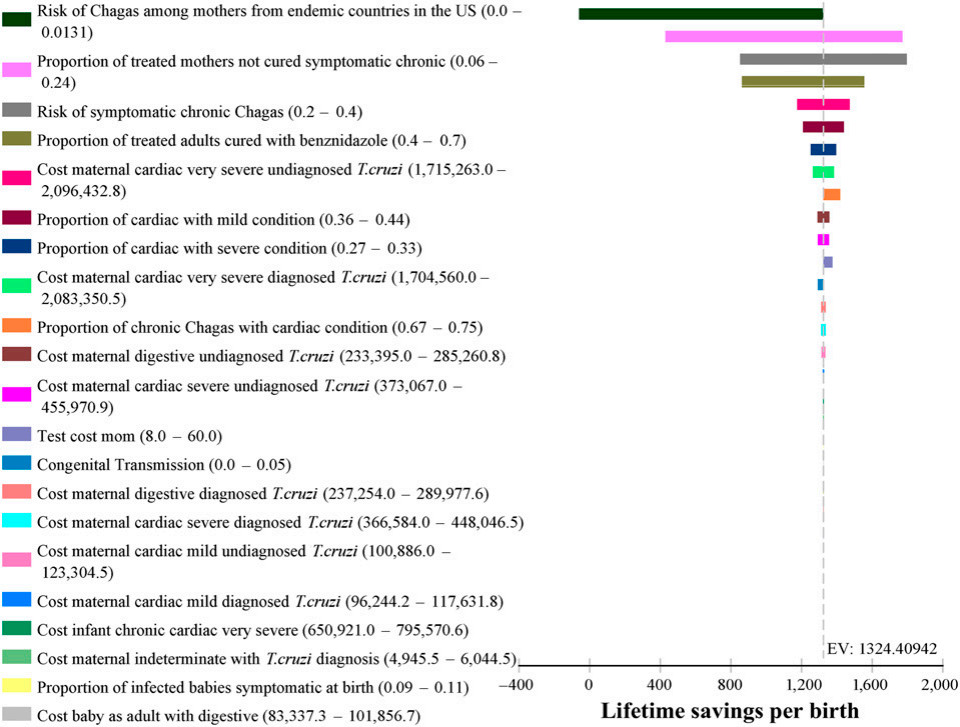
Incremental tornado analysis diagram. This figure appears in color at www.ajtmh.org.

Figure 2 shows the incremental tornado analysis diagram, assuming a $60 maternal diagnosis cost. The expected value of lifetime savings per birth indicated on the *x* axis is $1,324. The horizontal bars show, in descending order, the magnitude of the effect of changing the parameter values. Clearly, the only parameter that makes an important difference in the savings from the Screening option is maternal prevalence. Although we had anticipated that variation in the mother-to-child transmission rate would have an appreciable effect, it did not. Varying mother-to-child transmission from 0.0 to 0.05 had a trivial effect on the estimate of savings, ranking 13th among parameters and barely visible in Figure 2. Thus, even if maternal transmission is extremely low in the United States because of environmental or biological factors, it makes little difference in the large savings from screening. Maternal screening costs also had little impact on total costs.

A parameter that had a nontrivial effect on cost savings was the proportion of treated mothers who are not cured and become symptomatic. In the worst case scenario, with the highest probability of no cure (0.6) and the highest probability of being symptomatic (0.4), 24% of mothers face cardiac or digestive morbidity despite treatment. This combination affects both options, but, even so, savings per birth from the Screening option do not fall below $400.

## DISCUSSION

This decision analysis evaluated the lifetime societal costs and benefits of screening during pregnancy or at delivery for *T. cruzi* infection with the goal of treating infected mothers and infants to reduce or eliminate the risk of symptomatic Chagas disease. Those costs and benefits were compared with the lifetime costs of undiagnosed or late-diagnosed Chagas disease that would result from not screening mothers and infants. The results of the decision analysis demonstrate that the expected value, or per-birth cost, of the No Screening option is greater, generally substantially greater, than for the Screening option, over a broad range of screening costs and estimated prevalence. The total savings per birth year cohort of universal screening range from $426 to $634 million, for screening cost ranging from $60 to $8 per birth. We have not calculated the additional benefits of testing siblings and other family members. Those benefits, however, would amplify the results of this cost analysis.

There are an estimated 300,000 persons in the United States with Chagas disease.^4^ Using the standard estimation of the likelihood of developing symptoms (0.3), we could expect as many as 90,000 persons to develop cardiac or digestive symptoms out of the current population of infected persons in the United States. In the present analysis, we are estimating only the costs for pregnant women and their current pregnancy. The 63–315 infected infants born per year, if undiagnosed and untreated, face a 30% chance of developing disease symptoms. Thus, we can expect between 19 and 95 infants to develop cardiac or digestive symptoms of Chagas disease. Similarly, there would be 6,300 infected mothers giving birth each year, of whom 1,890 would be expected to develop cardiac or digestive symptoms of Chagas disease. These symptomatic infections in mothers and children are the source of more than $1 billion in lifetime costs of undiagnosed and untreated Chagas disease for each birth year cohort (see Table 4).

### Cost of the screening program.

The implementation costs of the screening program are far less than the costs attributed to the Screening scenario because that scenario includes the costs of cardiac and digestive symptoms of mothers who are diagnosed and treated but who are not cured (referred to in the section on incremental tornado analysis). Those costs for symptoms would result anyway in the No Screening scenario. The costs of the *T. cruzi* screening program alone would consist of testing for mothers, testing babies of infected mothers at birth, treating infected mothers and babies, and some additional cardiac monitoring for mothers in the indeterminate phase or with digestive conditions who are diagnosed as a result of the screening program but would not have been diagnosed under the No Screening scenario. The implementation cost of the screening program is shown in Table 6 for both targeted and universal screening at $60 and $8 screening cost. Universal screening at the new test cost would amount to about $66 million, about half of which is the screening cost and about half represents the costs of enhanced care for women with digestive or indeterminate conditions who would not have been identified in the No Screening scenario. The benefit ($634 million, the discounted present value of the savings) is thus almost 10 times the cost of the screening program for each birth year cohort.

**Table 6:** Implementation costs: targeted and universal screening

	Targeted (480,000 births)		Universal (4 million births)	
	At $60 per screen	At $8 per screen	At $60 per screen	At $8 per screen
Maternal screening	$28,800,000	$3,840,000	$240,000,000	$32,000,000
Newborn testing (6,300 × $400)	$2,520,000	$2,520,000	$2,520,000	$2,520,000
Additional monitoring for mothers, indeterminate phase 4,400 mothers × $5,495	$24,178,000	$24,178,000	$24,178,000	$24,178,000
Additional cardio monitoring for mothers, digestive symptoms 600 mothers × $12,250	$7,350,000	$7,350,000	$7,350,000	$7,350,000
Total implementation cost	$62,848,000	$37,888,000	$274,048,000	$66,048,000

### Targeted versus universal screening.

The decision between a targeted screening program and a universal program has financial implications, but logistical considerations may be paramount. A targeted program imposes an additional burden on obstetricians who would have to identify women from endemic areas among their patients. Profiling based on last name or other characteristic is unreliable and inappropriate, and so, doctors and physicians’ assistants would have to question new patients about the place of origin. Such an inquiry could make patients reluctant to seek prenatal care.

Universal prenatal and newborn screening is routine across the United States for numerous low-prevalence conditions, although the menu of tests may differ from state to state. HIV testing of pregnant women or newborns is routine in many states and has even been mandatory in some states. An estimated 8,500 HIV-infected women give birth each year, compared with an estimated 6,300 women with *T. cruzi* infection. The simplest strategy is universal screening for *T. cruzi* during prenatal care or at birth, even though we know that only about 12% of pregnant women are at risk. Universal screening is cost saving even at $60 per woman screened, but the new point-of-care test makes universal screening easier and more cost saving.

### Limitations.

Conservative use of the decision tree methodology, which we have followed, tends to underestimate the costs of the No Screening scenario, overestimate the costs of the Screening scenario, and thus underestimate the net savings of Screening. We are comparing No Screening to Screening, both with best practice in follow-up care in identified persons. That is, we assign costs to all cases discovered as if they would get pacemakers, transplants, annual cardiology checkups, or whatever the best care available would be. In the case of identified indeterminate chronic Chagas disease, for example, we assign the costs of biennial cardiology checkups for mothers up to age of life expectancy of 84 years. It is likely that many of the women would have stopped having biennial checkups after many years of good results.

The assumption that, without screening, symptomatic infants will be correctly diagnosed at birth, and that infected mothers and infants will be treated according to best practice, may be overly optimistic. Moreover, our estimate that 10% of infected newborns will be symptomatic may be high for the United States. Thus, in the model, all of these assumptions lead to substantially lower costs in the No Screening scenario. Consequently, the savings from Screening are understated in the results.

Ideally, in the Screening scenario, infants with negative test results born to infected mothers would be retested at 9 months of age, but loss to follow-up could reduce the impact of the program. Of course, the proportion of undiagnosed and untreated infants would still be far higher in the No Screening scenario because only infants symptomatic at birth would be diagnosed and treated.

For low-prevalence conditions, when diagnostic tests have < 100% specificity, the number of false positives could far exceed the true positives. This would be especially true with universal screening among the approximately 3.5 million women with almost no likelihood of exposure to infection. The new diagnostics, which would be suitable for universal screening, are reported in field trials to have 98.8% specificity in whole blood and 96.9% in serum.^30^ If actual specificity in the U.S. setting, for example, is 96%, the number of false positives among all 4 million births would be about 160,000. Some physicians, on consultation with the mother, might dismiss the results, but many would send them for confirmatory testing, which would increase costs. The estimated additional cost could be as high as $9.6 million, which although substantial, adds less than 15% to screening costs per se and reduces the savings from Screening versus No Screening by 1.5%, from $634 to $624 million per birth year cohort.

For comparison, rapid diagnostic tests (RDTs) for HIV are reported to have 99% specificity,^32,33^ but at least one RDT (First Response) had 100% specificity on whole blood but 82.86% specificity with serum specimen for HIV-1.^34^ Comparing results with serum specimen, the HIV test would generate far more false positives than the Chagas RDT with serum that was used for estimating cost stated earlier.

The decision analysis reports the costs and benefits (costs avoided) for each complete scenario. In the Screening scenario, all infected mothers would be treated, but only 60% of them would be cured. The medical costs and productivity losses from their Chagas symptoms are assigned to the Screening scenario, but those symptoms and costs would have occurred if the women had not been screened and a cure attempted. They are not a result of the Screening protocol but rather occur despite it.

We underestimate the savings of the Screening option because we do not include the savings for subsequent pregnancies for mothers treated as a result of screening.

Another factor is the low level of medical coverage of Hispanics in the United States, but the overestimation of access to care affects both scenarios. “In 2016, non-Hispanic Whites had the lowest uninsured rate among race and Hispanic origin groups, at 6.3%. The uninsured rates for Blacks and Asians were higher than for non-Hispanic Whites, at 10.5% and 7.6%, respectively. Hispanics had the highest uninsured rate, at 16.0%.”^35^ In the No Screening scenario, Hispanics may have lower medical costs but they would have higher rates of premature death (higher productivity losses) due to untreated cardiac and digestive conditions. In the No Screening scenario, there could be inadequate follow-up for women who are symptomatic or in the indeterminate phase if they are uninsured and, particularly, if they are undocumented and fear deportation.

Because benznidazole has been approved for commercial sale in the United States, it will eventually mean additional costs for the Screening option. It would be speculative at this time to choose a price to include in the analysis. We expect that the cost will not be exorbitant, given that the FDA-approved product is made by a company that has a corporate social responsibility partner devoted to access to treatment of Chagas disease (https://www.dndi.org/2016/media-centre/press-releases/partnership-register-benzindazole-usa-latinamerica/).

It is projected that successful domestic vector control programs in endemic areas of Latin America and prenatal screening programs would ultimately obviate the need for the proposed program.^1^ It is, in the best case, a self-limiting program. This model, then, does not represent longer term future costs because Chagas disease could be virtually eliminated in human populations. A strategy to speed up that process would include a test-and-treat program for young Hispanics from endemic areas. Treating young women before pregnancy can reduce their own morbidity and prevent transmission to future children.^19,20^ Treating young men for their own future health would be ethically appropriate as well.

## CONCLUSION

Maternal screening, along with infant testing and maternal and infant treatment as indicated, has the potential to reduce disability and death from Chagas disease in the United States substantially. Cost savings from a societal perspective from such a program would be outstanding, and the results are robust across a wide range of prevalence, rates of mother-to-child transmission, and screening costs, as well as all other clinical and cost parameters. Sensitivity analysis in our model demonstrates that, even at current testing costs, maternal screening and follow-up infant testing and treatment as indicated is cost saving for maternal prevalence as low as 0.057% and for mother-to-child transmission probability as low as 0.001%. With the new point-of-care test, universal screening is cost saving for prevalence as low as 0.008% of pregnant women. The human and economic cost of Chagas disease is very great, even in a country with low prevalence and low transmission. Lifetime societal costs, including direct medical costs and productivity loss due to morbidity and premature mortality, are nearly 10 times the cost of implementing a universal maternal screening program. This analysis supports adding testing for *T. cruzi* infection to routine screening in pregnancy or at the time of birth in the United States.

## Supplementary Material

Supplemental Text, Tables, and Figures.
