# Supplementary figures and images for: Congenital Chagas Disease in the United States: Cost Savings through Maternal Screening
Source: Am J Trop Med Hyg. 2018 Apr 30;98(6):1733–42. doi: 10.4269/ajtmh.17-0818 (PMC6086189; doi:10.4269/ajtmh.17-0818)

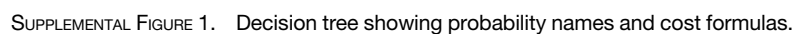

Supplement: Supplementary file 1 [file tpmd170818.SD1.pdf]
