# Supplementary material for: Congenital Chagas Disease in the United States: Cost Savings through Maternal Screening
Source: Am J Trop Med Hyg. 2018 Apr 30;98(6):1733–42. doi: 10.4269/ajtmh.17-0818 (PMC6086189; doi:10.4269/ajtmh.17-0818)
Supplement: Supplementary file 2 [file tpmd170818.SD2.pdf]

S1. Detailed Methodology, Calculations, and Sources

Stillwaggon E, Perez-Zetune V, Bialek S, and Montgomery S. Congenital Chagas disease in the United States: cost savings through maternal screening.
This supplement contains detailed explanations of the decision-tree methodology, the calculation of parameter estimates, and the sources of data used in the article.

We explain the choice of point estimates and ranges for probabilities used in the decision tree, the catalogue of treatment protocols proposed in the medical literature, the derivation of treatment costs, based on Medicare allowances and other sources, and estimates of productivity losses based on expected morbidity and mortality.

TABLE 1

Table 1 (of the main article) shows the probabilities of maternal infection (prevalence among women of child-bearing age), maternal transmission, conversion from indeterminate form to symptomatic form, and other risks of morbidity and mortality. Point estimates and ranges are derived from the literature indicated in the source column.

Maternal prevalence: there is a wide range of estimates for maternal prevalence. We use Bern and Montgomery[1] for the point estimate, but, considering the lower prevalence reported in di Pentima *et al.*, which found 0.4% prevalence among mothers at delivery in a hospital in Texas[2] and Edwards *et al*, which found a 0.25% prevalence among 4,000 predominantly Hispanic women in southern Texas[3], we extended the lower bound in the sensitivity analysis to 0.0. In sensitivity analysis, we found that both the di Pentima and Edwards estimates are well above the threshold for which Screening is cost-saving (0.057%).

Mother-to-child-transmission (MTCT): there is again a wide range of estimates. We use the mid-range estimate of 5%, but our sensitivity analysis shows that with maternal transmission as low as 0.001% the Screening option is cost-saving. The variation reported in MTCT may be due to higher transmission from mothers in highly endemic areas who are exposed to reinfection and thus have higher parasitemia than mothers in non-endemic areas.[4] On the other hand, higher parasitemia and higher transmission risk have been reported among mothers not exposed to reinfection, possibly due to loss of immunity.[5]

Risk of symptomatic Chagas: estimates of the probability of cardiac or digestive symptoms, or both, occurring in infected persons, generally 10 to 20 years after infection, range from 20% to 40%,[6, 7] but 30% is commonly cited.[8-10]

Cardiac and digestive symptoms: available data suggest that for approximately 2/3 of persons with Chagas disease morbidity, the symptoms will be cardiac, and for about 1/3 the morbidity will affect the digestive tract. Some persons will have both cardiac and digestive forms.[6]

Cardiac, mild, severe, very severe: it is estimated that 60% of Chagas cardiac patients die of heart disease,[11] and we classify the remaining 40% as having mild cardiac complications. Based on the finding of a 4% annual mortality rate of persons with cardiac Chagas disease,[11] we estimated a survival curve for the 60% of persons with fatal cardiac complications. Half of those persons are projected to die within 16 years and we categorize them as having very severe Chagas disease and the remainder as having severe cardiac Chagas disease and surviving 26 years after onset of symptoms. Life expectancy for persons with mild cardiac conditions is estimated to be 65 years.

Risk of baby symptomatic at birth: data are scarce for the proportion of *T. cruzi*-infected infants symptomatic at birth. For newborns, symptoms can be non-specific and not identified as caused by *T. cruzi*. Symptoms may also be more common among newborns of highly parasitemic mothers in highly endemic areas. Several sources report 10% to 40% of newborns symptomatic, but the higher estimates may be for highly endemic areas where mothers are also poorly nourished and have other pathologies.[4, 12] Based on the estimate of 63 to 315 infected babies per year in the United States[1] and the identification of only 2 babies in the United States due to symptoms at birth,[12] we use the lower bound, 10%, which may still be high for the United States. As noted in the article, the effect of using a high estimate for symptomatic infection at birth is to understate costs in the No-Screening scenario and thus understate the savings from the Screening option.

Distribution of symptomatic at birth: although few data exist on infants infected with *T. cruzi*, infant mortality is reported between 2% and 20% [13-15] for those infected, and we used the most conservative estimate for the analysis. Of the infected infants, up to 40% are symptomatic at birth,[4, 7] of whom 5% may die; thus overall the infant mortality due to Chagas disease is 2%, the lower bound in the literature. Symptomatic infants who do survive past birth could develop severe symptoms such as respiratory complications, myocarditis, and meningoencephalitis.[4, 14, 15] In Bolivia, Torrico *et al*.[15] found that 42–54% of symptomatic infants display at least one severe symptom, and we chose the point estimate of 45% of infants. Finally, 50% of the symptomatic infants are classified as mildly symptomatic, displaying fever, prematurity, low birth weight, and a low Apgar score. The distribution of severity of symptoms is conditional on the estimate of 10% of infected infants being symptomatic.

Mother cured: there are a number of sources that report parasite clearance rates for adults who are without apparent cardiac or digestive damage,[7] as might be expected for mothers at age 27, the average age for Hispanic mothers giving birth.[16]

TABLE 2

Table 2 (of the main article) lists all of the unit costs used in the decision tree, including costs of testing, chemotherapy for infants and mothers, costs of hospital care for symptomatic newborns, and costs of interventions for cardiac and digestive tract morbidity.

Unit costs were taken from sources reporting Medicare payments, either government sources themselves or sources that use government-allowed payments, as indicated in the Source column in Table 2.

For costs of inpatient procedures we used http://www.medicarehelp.org, which lists for each state the average of charges for a procedure and the Medicare payment for the procedure in 2016. The Medicare payment is considered a reflection of the real cost. We examined the charges and costs for a number of states and chose Maryland because it had the highest cost-to-charge ratio, which is confirmed in Haddix *et al*.,[17] Appendix F, derived from the *Federal Register*. The National Capital Area, of which Maryland is a part, has among the nation's highest population of Bolivian origin, who would be expected to have the highest rates of Chagas disease. **Table S1** shows the variation among selected locations for average costs and charges.

Table S1. Costs and charges for selected states

State	Procedure	Medicare payment	Average Charge	Ratio
Maryland	Pacemaker without complications	$19,577	$20,753	0.94
Maryland	Pacemaker with complications	$24,882	$26,454	0.94
Pennsylvania	Pacemaker without complications	$13,393	$60,306	0.22
Pennsylvania	Pacemaker with complications	$17,545	$80,782	0.22
Texas	Pacemaker without complications	$13,164	$60,525	0.22
Texas	Pacemaker with complications	$17,081	$76,114	0.22
District of Columbia	Pacemaker without complications	$15,797	$44,150	0.36
District of Columbia	Pacemaker with complications	$22,170	$72,724	0.30

Source: http://www.medicarehelp.org

Pacemaker: we used the average of costs with and without complications in Maryland for our pacemaker estimate from Table S1 above.

Heart transplant: we used the total of all charges calculated in Bentley[18] and then applied the cost-to-charge ratio for Maryland urban hospitals, 0.759, reported in Haddix *et al*.[17]

Fundoplication: costs for fundoplication, a treatment for severe esophageal morbidity, are derived from the cost of "Esophagitis Gastroent and Misc Digest Disorders with Major complications," found at http://www.medicarehelp.org for Maryland Medicare payments.

Colon resection: costs for surgery for severe megacolon are derived from the cost for "Major small and large bowel procedures with major complications," reported at http://www.medicarehelp.org for Maryland Medicare payments.

Amiodarone: used for arrhythmia. The dosage begins high to arrest the arrhythmia and then the patient is transitioned to a maintenance dose. Consequently there is a cost for the first year and a lower cost for subsequent years. The calculations are as follows:
"Loading dose: 800 to 1600 mg orally per day for 1 to 3 weeks (occasionally longer) until adequate arrhythmia control is achieved or if side effects become prominent"
https://www.drugs.com/dosage/amiodarone.html#Usual_Adult_Dose_for_Arrhythmias. The same information is found at: http://reference.medscape.com/drug/pacerone-cordarone-amiodarone-342296.
Drug costs are found at: https://www.medicaid.gov/medicaid/prescription-drugs/pharmacy-pricing/index.html and https://data.medicaid.gov/Drug-Prices/NADAC-as-of-2017-01-18/e97y-sprb.
3 tablets x 400 mg x 14 days = 3 x $3.32 x 14 = $140 ($139.44) (loading)
2 x 400 mg x 30 d = 2 x $3.32 x 30 = $200 ($199.20) (adjustment)
2 x 200 mg x 320 d = 2 x $0.13 x 320 = $85 ($83.20) (maintenance)
Total for first year = $425 ($421.84)
2 tablets per day x 200 mg x number of surviving years (27 to 65 or 30 to 65) = 2 x $0.13 x 365 x years = $100 per year ($94.90).

Esophageal relaxant: Sildenafil is prescribed for esophageal spasms. Dosage is one pill per day at $0.44 per pill = $0.44 x 365 = $160.60 = $160.
http://www.mayoclinic.org/diseases-conditions/esophageal-spasms/basics/treatment/con-20025653

Drug costs are found at: https://www.medicaid.gov/medicaid/prescription-drugs/pharmacy-pricing/index.html and https://data.medicaid.gov/Drug-Prices/NADAC-as-of-2017-01-18/e97y-sprb.

Laxative: "Usual Adult Dose for Constipation, Oral: 50 to 400 mg (using any of the salt forms) orally administered in 1 to 4 equally divided doses each day. https://www.drugs.com/dosage/docusate.html and https://www.drugs.com/pro/docusate-sodium.html.
Drug costs are found at: https://www.medicaid.gov/medicaid/prescription-drugs/pharmacy-pricing/index.html and https://data.medicaid.gov/Drug-Prices/NADAC-as-of-2017-01-18/e97y-sprb.
3 x 100 mg tablet per day = 3x $0.03 x 365 = $32.85 = $33.

TABLE 3

Table 3 (of the main article) summarizes the present value of costs of typical protocols for possible outcomes, including the indeterminate form and cardiac and digestive complications of the chronic stage, including productivity losses due to premature mortality. The following discussion provides detailed explanation of the components of costs listed in Table 3 and summarized later.

Productivity losses: for mothers and for untreated babies who develop symptoms as adults, we calculate losses due to mortality based on on-line data referenced in Grosse *et al*.[19] and use the productivity loss for males and females averaged to avoid the devaluation of women's work, as suggested in Grosse *et al*. The data available on line include losses for every year of age and for discount rates from 1% to 10%, at http://www.johnwardeconomics.com/, and the specific link for the tables of losses is https://drive.google.com/file/d/0B73RAsHdW6D3UGs4YTRiTHlQUU0/view.

Grosse *et al*.[19] calculate the productivity losses including the assumption of a 1% gain in productivity (and wages) over the coming decades because that had been the case until fairly recently. Productivity gains and wage gains, however, have leveled off and so, as suggested in the article, we use a 4% discount rate in order to correct for the 1% productivity gain included in the cited table to achieve the 3% discount rate that is conventionally used in health economics analyses. We do not inflate losses from the 2007 data because of the flat wage gains in the past decade. We are grateful to Scott Grosse and Martin Meltzer of CDC for a very helpful conversation on this issue.

The national average earnings that are used to calculate the productivity losses in Grosse *et al*.[19] are higher than average Hispanic wages, which are 0.69 of non-Hispanic wages for men and 0.58 of non-Hispanic wages for women (http://www.pewresearch.org/fact-tank/2016/07/01/racial-gender-wage-gaps-persist-in-u-s-despite-some-progress). Labor-force participation, on the other hand, is higher for Hispanic men (76.1%) than for non-Hispanic white men (68.4%), whereas labor-force participation rates for Hispanic and non-Hispanic white women are almost equal, 56% and 56.9% respectively (https://www.bls.gov/emp/ep_table_303.htm). While actual earnings loss for Hispanics might be less than the national average, we use the national figures for productivity loss to avoid devaluing Hispanic lives.

Productivity loss for neonatal death in 2016: the loss in the on-line table for newborns at 0% discount rate is $4,309,701, and $759,393 at 4% discount rate https://drive.google.com/file/d/0B73RAsHdW6D3UGs4YTRiTHlQUU0/view.

Table S2 shows the present value of productivity losses for premature mortality of mothers and babies based on projected year of death.

Table S2. Age at death for various conditions and productivity loss

**Person**<br>**Condition**	**Age at death**	**Year of death**	**Present value of productivity loss**
Baby at birth	0	2016	$ 759,393
Baby as adult			
Cardiac mild	65	2081	$ 31,899
Cardiac severe	53	2069	$ 130,130
Cardiac very severe	43	2059	$ 327,406
Digestive	65/53*	2081/2069	$ 81,015
Mother			
Cardiac mild	65	2054	$ 91,978
Cardiac severe	53	2042	$ 375,212
Cardiac very severe	43	2032	$ 944,033
Digestive	65/53*	2054/2042	$ 233,595
*All costs for digestive morbidity are averaged because of paucity of data on the distribution of esophageal and colonic disease and between mild and severe cases. Half of persons affected have a life expectancy of 65 and half have a life expectancy of 53.			

Table S3 summarizes in detail the protocol for each condition for mother and baby and the costs derived for each.

Maternal age at screening or onset: We use the average maternal age for Hispanic births of 27 (https://www.cdc.gov/nchs/data/databriefs/db232.htm). We assume that mothers who are screened will be diagnosed in prenatal care or at delivery. Those who are in the **Indeterminate** phase will begin biennial visits to a general practitioner, consisting of a medical exam and electrocardiogram (ECG) with stress test. There will be no productivity loss in Indeterminate phase. Mothers who are not screened, but who are in the Indeterminate phase, will not know they are infected and will receive no follow-up care, unless their babies are born symptomatic. In the latter case, we assume best practice: both baby and mother will be diagnosed and treated. If the mother is not cured, her prognosis follows that of non-treated mothers.

Mothers who are not diagnosed with *T. cruzi* infection, because they are in the No Screening branch and their babies are asymptomatic if infected, who develop symptoms of Chagas disease can follow one of several paths. Symptoms (and costs) begin with an acute episode of cardiac or digestive symptoms, at which time care and outcomes begin for the level and type of condition. In the absence of screening, a person would visit a doctor or the emergency room perhaps multiple times before receiving a diagnosis of heart failure or arrhythmia and then be prescribed medication or receive a pacemaker or transplant. Similarly, determining an appropriate treatment for digestive disorders would require visits to medical practitioners, or in severe cases, the emergency room. Infected babies in the No Screening branch who are in the Indeterminate phase will receive no care as adults. Infected babies who have cardiac or digestive symptoms as adults will follow the same protocol as mothers who have symptoms but are not diagnosed with *T. cruzi*.

Table S3: Detailed conditions, protocols, and costs

Condition	*Protocol*	*Frequency*	*Costs (present value) in US$*		
			Mothers with diagnosis	***Mothers without diagnosis***	***Undiagnosed babies as adults***
**Chronic, indeterminate**	General practitioner	Biennial to age 84	2,337	No care	No care
	ECG + stress test	Biennial to age 84	3,097	No care	No care
	Chest x-ray	Onset year	61	No care	No care
	**Total**		**5,495**	**0**	**0**
	Name in tree		*M_indet*		
**Chronic cardiac, mild**	Cardiologist exam	Annual to age 65	7,118	7,118	2,807
	ECG + stress test	Annual to age 65	5,168	5,168	2,038
	Amiodarone, first year	Onset year	425	425	175
	Amiodarone, annual	Annual to age 65	2,249	2,249	885
	Mild cardiac onset	Onset year	0	5,157	2,125
	Productivity loss	At age 65	91,978	91,978	31,899
	**Total**		**106,938**	**112,095**	**39,929**
	Name in tree		*M_Car_Mild_Dx*	*M_Car_Mild_NoDx*	*Inf_Car_Mild*
**Chronic cardiac, severe**	Cardiologist exam	Annual to age 53	5,720	5,720	2,178
	ECG + stress test	Annual to age 53	4,153	4,153	1,581
	Pacemaker	Onset year	22,230	22,230	9,158
	Severe cardiac onset	Onset year	0	7,204	2,968
	Productivity loss	At age 53	375,212	375,212	130,130
	**Total**		**407,315**	**414,519**	**146,015**
	Name in tree		*M_Car_Sev_Dx*	*M_Car_Sev_NoDx*	*Inf_Car_Sev*
**Chronic cardiac, very severe**	Cardiologist exam	Annual to age 43	4,109	4,109	1,452
	ECG + stress test	Annual to age 43	2,983	2,983	1,055
	Heart transplant	Onset year	942,830	942,830	388,433
	Very severe cardiac onset	Onset year	0	11,893	4,900
	Productivity loss	At age 43	944,033	944,033	327,406
	**Total**		**1,893,955**	**1,905,848**	**723,246**
	Name in tree		*M_Car_VerySev_Dx*	*M_Car_VerySev_NoDx*	*Inf_Car_VerySev*

Esophageal, Mild	Gastroenterologist	Annual to age 65	7,283	7,283	2,872
	Esophageal relaxants	Annual to age 65	3,759	3,759	1,482
	Cardiologist exam	Annual to age 65	7,118	No care	No care
	ECG + stress test	Annual to age 65	5,168	No care	No care
	Esophagitis onset	Onset year	0	6,707	2,763
	Productivity loss	At age 65	91,978	91,978	31,899
	Chest x-ray	Onset year	61	No care	No care
	Subtotal		115,367	109,727	39,016
Esophageal, severe	Gastroenterologist	Annual to age 53	5,852	5,852	2,228
	Fundoplication	Onset year	11 234	11 234	4,628
	Cardiologist exam	Annual to age 53	5,720	No care	No care
	ECG + stress test	Annual to age 53	4,153	No care	No care
	Esophagitis onset	Onset year	0	6,707	2,763
	Productivity loss	At age 53	375,212	375,212	130,130
	Chest x-ray	Onset year	61	No care	No care
	Subtotal		390,998	387,771	139,749
Colon, Mild	Gastroenterologist	Annual to age 65	7,283	7,283	2,872
	Laxatives	Annual to age 65	775	775	306
	Cardiologist exam	Annual to age 65	7,118	No care	No care
	ECG + stress test	Annual to age 65	5,168	No care	No care
	Colon mild onset	Onset year	0	5,916	2,437
	Productivity loss	At age 65	91,978	91,978	31,899
	Chest x-ray	Onset year	61	No care	No care
	Subtotal		112,383	105,952	37,514
Colon, severe	Gastroenterologist	Annual to age 53	5,852	5,852	2,228
	Colon resection	Onset year	44,718	44,718	18,423
	Cardiologist exam	Annual to age 53	5,720	No care	No care
	ECG + stress test	Annual to age 53	4,153	No care	No care
	Colon severe onset	Onset year	0	8,079	3,328
	Productivity loss	At age 53	375,212	375,212	130,130
	Chest x-ray	Onset year	61	No care	No care
	Subtotal		435,716	433,861	154,109
**Digestive**	**Average**		**263,616**	**259,328**	**92,597**
	Name in tree		*M_Digest_Dx*	*M_Digest_NoDx*	*Inf_Digest*

Treatment plans for every individual will undoubtedly vary, and the standard treatment here does not incorporate co-morbidities. We outline here a typical package of care for modeling purposes.

Mild cardiac entails a mild onset episode and then annual visits with a cardiologist, annual ECG with stress test, and amiodarone, plus a productivity loss for death at 65. Costs for the mild cardiac onset episode are taken from “Cardiac arrhythmia and conduction disorders no complications” for Maryland, in http://www.medicarehelp.org, the same source that was used for inpatient procedures listed above.

Severe cardiac entails a severe onset episode and then annual visits with a cardiologist, annual ECG with stress test, and a pacemaker, plus a productivity loss for death at 53. Costs for the severe cardiac onset episode are taken from “Cardiac arrhythmia and conduction disorders with complications” for Maryland, in http://www.medicarehelp.org.

Very severe cardiac entails a very severe onset episode and then annual visits with a cardiologist, annual ECG with stress test, and a heart transplant, plus a productivity loss for death at 43. Costs for the very severe cardiac onset episode are taken from “Cardiac arrhythmia and conduction disorders with major complications” for Maryland, in http://www.medicarehelp.org.

Digestive symptoms can be esophageal or colonic. Published studies provide sufficient data on chronic cardiac Chagas disease to estimate the percentage of persons with, and thus the costs of, mild, severe, and very severe cardiac complications. There are few published data, however, on the proportions of persons with different kinds of digestive complications. Consequently, we estimated the lifetime costs of treatment for mild and severe megacolon and megaesophagus and used the average of those costs for all persons with digestive complications. In the case of mothers who are diagnosed with *T. cruzi*, either because of screening or because they gave birth to a symptomatic infected baby, if they have digestive symptoms, they will also have a cardiologist exam and ECG with stress test annually. For mothers without *T. cruzi* diagnosis, it is assumed that their digestive problems will not be identified as Chagas disease. Consequently, they will have onset episodes that will entail doctor visits and possible emergency room visits until they receive appropriate symptomatic care.

Costs for the onset episode for both mild and severe esophageal symptoms are taken from “Esophagitis Gastroent and Misc Digest Disorders No complications” for Maryland, in http://www.medicarehelp.org.

Costs for the onset episode for mild colon symptoms are taken from “GI Obstruction no complications” for Maryland, in http://www.medicarehelp.org.

Costs for the onset episode of severe colon symptoms are taken from “GI Obstruction with complications” for Maryland, in http://www.medicarehelp.org.

Annual number of births in United States to women from endemic regions – We calculate back from Bern and Montgomery who estimate 63 to 315 births annually of congenitally infected babies. They use a maternal transmission rate of 1% to 5% and a maternal prevalence of 1.31% based on US population of persons from endemic countries. Thus they are estimating 480,916 births annually to women from endemic countries.[1]

What the lifetime costs represent

While the decision tree accurately portrays the expected value of costs for each scenario, it may not be an intuitively accessible way of seeing the results. To understand the costs, we can look at specific outcomes. Each terminal node in Figure S1 (Decision tree before calculation) displays a formula for the total cost of

the outcomes for each mother-child pair. Terminal node #17 in the No Screening scenario, for example, displays the formula:

CostSympMild+Dx_baby+Dx_mom+Rx_baby+Rx_mom+M_indet

which indicates that the infant was treated for mild symptoms at birth, the baby and mother were diagnosed and treated, the baby was cured, but the mother remained in the indeterminate phase and thus there were lifetime costs of monitoring the mother's condition. Present value of lifetime costs for that outcome would be $8,555, shown after calculation in Figure S2.

Terminal node #59, Figure S1, also in the No Screening scenario, displays the formula:

Inf_Digest + M_Car_VerySev

for the case in which the newborn is asymptomatic and so neither the mother nor the child is diagnosed or treated. The infant develops digestive symptoms later in life and the mother develops very severe cardiac symptoms. The total economic cost for this outcome is $1,998,445, shown after calculation in Figure S2. The probability of this outcome is 0.000004, and so with more than 480,000 births per year (in the targeted scenario), we could expect two mother-child pairs per year with this combination of symptoms and this level of lifetime costs, or approximately $4 million.

References

1. Bern C, Montgomery SP, 2009. An estimate of the burden of Chagas disease in the United States. *Clin Infect Dis 49*: e52-4.
2. Di Pentima MC, Hwang LY, Skeeter CM, Edwards MS, 1999. Prevalence of antibody to *Trypanosoma cruzi* in pregnant Hispanic women in Houston. *Clin Infect Dis 28*: 1281-5.
3. Edwards MS, Rench MA, Todd CW, Czaicki N, Steurer FJ, Bern C, Montgomery SP, 2015. Perinatal screening for Chagas disease in southern Texas. *J Pediatric Infect Dis Soc 4*: 67-70.
4. Norman FF, Lopez-Velez R, 2014. Mother-to-child transmission of *Trypanosoma cruzi* infection (Chagas disease): a neglected problem. *Trans R Soc Trop Med Hyg 108*: 388-90.
5. Rendell VR, Gilman RH, Valencia E, Galdos-Cardenas G, Verastegui M, Sanchez L, Acosta J, Sanchez G, Ferrufino L, LaFuente C, Abastoflor Mdel C, Colanzi R, Bern C, 2015. *Trypanosoma cruzi*-infected pregnant women without vector exposure have higher parasitemia levels: implications for congenital transmission risk. *PLoS One 10*: e0119527.
6. Rassi A, Jr., Rassi A, Marin-Neto JA, 2010. Chagas disease. *Lancet 375*: 1388-402.
7. Sicuri E, Munoz J, Pinazo MJ, Posada E, Sanchez J, Alonso PL, Gascon J, 2011. Economic evaluation of Chagas disease screening of pregnant Latin American women and of their infants in a non endemic area. *Acta Trop 118*: 110-7.
8. Hotez PJ, Dumonteil E, Betancourt Cravioto M, Bottazzi ME, Tapia-Conyer R, Meymandi S, Karunakara U, Ribeiro I, Cohen RM, Pecoul B, 2013. An unfolding tragedy of Chagas disease in North America. *PLoS Negl Trop Dis 7*: e2300.
9. Klein N, Hurwitz I, Durvasula R, 2012. Globalization of Chagas disease: a growing concern in nonendemic countries. *Epidemiology Research International 2012*: 12.
10. Sanchez Negrette O, Mora MC, Basombrio MA, 2005. High prevalence of congenital *Trypanosoma cruzi* infection and family clustering in Salta, Argentina. *Pediatrics 115*: e668-72.
11. Rassi A, Jr., 2007. Implantable cardioverter-defibrillators in patients with Chagas heart disease: misperceptions, many questions and the urgent need for a randomized clinical trial. *J Cardiovasc Electrophysiol 18*: 1241-3.
12. Centers for Disease C, Prevention, 2012. Congenital transmission of Chagas disease - Virginia, 2010. *MMWR Morb Mortal Wkly Rep 61*: 477-9.
13. Bern C, Kjos S, Yabsley MJ, Montgomery SP, 2011. *Trypanosoma cruzi* and Chagas' Disease in the United States. *Clin Microbiol Rev 24*: 655-81.
14. Ramsey JM, Elizondo-Cano M, Sanchez-Gonzalez G, Pena-Nieves A, Figueroa-Lara A, 2014. Opportunity cost for early treatment of Chagas disease in Mexico. *PLoS Negl Trop Dis 8*: e2776.

15. Torrico F, Alonso-Vega C, Suarez E, Rodriguez P, Torrico MC, Dramaix M, Truyens C, Carlier Y, 2004. Maternal *Trypanosoma cruzi* infection, pregnancy outcome, morbidity, and mortality of congenitally infected and non-infected newborns in Bolivia. *Am J Trop Med Hyg 70*: 201-9.
16. Martin JA, Hamilton BE, Osterman MJK, Curtin SC, Mathews TJ, 2015. Births: Final data for 2013. National vital statistics reports. Hyattsville MD: National Center for Health Statistics.
17. Haddix AC, Teutsch SM, Corso PS, 2003. Prevention Effectiveness: A Guide to Decision Analysis and Economic Evaluation. New York: Oxford University Press.
18. Bentley TS, 2014. 2014 U.S. organ and tissue transplant cost estimates and discussion. Milliman Research Report. Brookfield WI: Milliman, Inc.
19. Grosse SD, Krueger KV, Mvundura M, 2009. Economic productivity by age and sex: 2007 estimates for the United States. *Med Care 47*: S94-103.
